# Mercury in Fish and Human Hair and Estimated Dietary Intake in a Riverside Community of the Madeira River Basin in the Brazilian Amazon

**DOI:** 10.3390/toxics12030208

**Published:** 2024-03-09

**Authors:** Thayson Araujo Canela, Lucas Cabrera Monteiro, Cássio da Silva Cabral, Fábio da Silva Ximenes, Iuri Aparecida da Silva Oliveira, José Vicente Elias Bernardi, Ronaldo de Almeida, Wanderley Rodrigues Bastos

**Affiliations:** 1 Laboratório de Biogeoquímica Ambiental Wolfgang C. Pfeiffer, Universidade Federal de Rondônia, Porto Velho 76801-059, Rondônia, Brazil; tac84454847@gmail.com (T.A.C.); cassio.unir@gmail.com (C.d.S.C.); 44711@mpro.mp.br (F.d.S.X.); iuria.oliveira@gmail.com (I.A.d.S.O.); bastoswr@unir.br (W.R.B.); 2 Programa de Pós-Graduação em Ecologia, Instituto de Ciências Biológicas, Universidade de Brasília, Distrito Federal, Brasília 73345-010, Goiás, Brazil; lcabreramonteiro@gmail.com; 3 Programa de Pós-Graduação em Desenvolvimento Regional e Meio Ambiente, Universidade Federal de Rondônia, Porto Velho 76801-059, Rondônia, Brazil; 4 Laboratório de Geoestatística e Geodésia, Faculdade UnB Planaltina, Universidade de Brasília, Distrito Federal, Planaltina 73345-010, Goiás, Brazil; bernardi@unb.br

**Keywords:** Hg emissions, EDI, PTWI, hazard quotient, water, ASGM

## Abstract

Mercury (Hg) is a chemical element that poses risks to human health due to its high toxicity and environmental persistence. We determined the total Hg (THg) and methyl Hg (MeHg) concentrations in hair samples from residents of the Demarcação District (Porto Velho, Rondônia) in the Brazilian Amazon, as well as in water and fish samples, to evaluate factors influencing human exposure. The average THg concentration in human hair was 7.86 ± 6.78 mg kg^−1^ and it was significantly higher in men, with an increasing trend related to age. There was no significant difference between female age groups. Human exposure to Hg through water was negligible compared to fish consumption. The average weekly intake estimates in the community varied between 1.54 and 4.62 μg kg^−1^, substantially higher than the recommended limit. The fish species with the highest amounts safe for daily consumption were herbivores and detritivores. Our results contribute to an understanding of how exposure to Hg affects the health of riverside populations and provide insights for new research to develop methods to mitigate such exposure and thus improve the quality of life of Amazonian people.

## 1. Introduction

Mercury (Hg) is a chemical element widely distributed in the environment, mobilized naturally through volatilization, marine aerosols, and volcanic eruptions [1]. However, the intensification of anthropogenic activities after the industrial revolution significantly increased Hg emissions [2], altering the element’s environmental distribution on a global scale [3]. In South America, industrial expansion from the 1970s onward has resulted in a significant increase in emissions of elemental Hg (Hg^0^) into the atmosphere, with approximately 90% being rapidly oxidized, converted into inorganic Hg (Hg^2+^), and deposited in aquatic and terrestrial ecosystems [4].

Currently, artisanal and small-scale gold mining (ASGM) is the main anthropogenic source of Hg in tropical regions [5], especially in the Amazon. In Brazil, it is estimated that 10 to 50 t of Hg derived from ASGM is emitted into aquatic and terrestrial ecosystems per year [6]. This environmental impact is particularly evident in the Amazon [7], where metallic Hg^0^ is widely used in mining to separate gold granules from sediments (amalgams) [8]. Losses of Hg^0^ during this separation process occur mainly due to the volatilization of Hg^0^ through burning the amalgam, in addition to the direct emission of residual Hg associated with mining tailings into aquatic ecosystems [9,10], although distillers are currently used in gold–mercury (Au-Hg) separation processes, especially in authorized ASGM. Additionally, natural Hg concentrations are relatively high in the Brazilian Amazon due to rock weathering and continuous atmospheric deposition [11]. Increasing deforestation and large-scale forest fires in the region intensify the remobilization of Hg^2+^ retained in terrestrial ecosystems and, consequently, its transport to aquatic ecosystems [5,12].

The Hg^0^ emitted into aquatic ecosystems is oxidized through complexation with organic and inorganic compounds and photochemical reactions [13,14]. However, Hg^2+^ is readily available for conversion to methylmercury (CH_3_Hg or MeHg) by bottom sediment microbiota, mainly sulfate- and iron-reducing bacteria and methanogenic archaebacteria [15]. MeHg is a highly bioavailable organomercury compound with great affinity for sulfur-rich proteins [16] capable of accumulating in the tissues of organisms (bioaccumulation) so that its concentration increases progressively according to the organism’s trophic level (biomagnification) [17]. In this sense, the MeHg available in aquatic ecosystems is accumulated by primary producers, such as phytoplankton and macrophytes, and transferred through trophic interactions to the fish that occupy the top of the food chain [18]. As a result, MeHg:THg ratios in fish are high (up to 90%) [19], representing the main route of non-occupational human exposure to Hg [20].

In the Amazon, fish consumption is the main source of protein and the main route of exposure to MeHg in riverside populations [21,22,23], often exceeding the safe limits established by regulatory agencies [24,25,26,27]. In humans, approximately 15% of Hg^2+^ is absorbed from the gastrointestinal tract and accumulated in the liver and kidneys, while the majority is excreted through feces and urine [28,29]. On the other hand, due to the affinity of MeHg for compounds from the thiol group (-SH) present in proteins, up to 95% of the MeHg consumed by humans is absorbed by the gastrointestinal tract and distributed in the body through the bloodstream, accumulating in the liver, kidneys, brain, and hair [29,30]. In this sense, the binding of MeHg with compounds from the keratin thiol group favors its accumulation in hair [16,31], thus representing an important biomarker for evaluating dietary exposure to Hg [32].

The high toxicity and high environmental persistence of Hg prompted the issuance of the Minamata Convention, an international treaty whose main objectives are to control Hg emission sources and mitigate risks to human health and the environment associated with Hg exposure [33]. This exposure can result in neuroinflammation and changes in metabolic pathways, oxidative stress, and neural signaling [34]. The risks are greater in pregnant women, in whom the transfer of Hg to the fetus through the placenta and umbilical cord [35,36] can cause congenital malformations and developmental delays in the fetus [37]. Additionally, Hg can also be transferred to children through breastfeeding [38,39,40]. Therefore, several indices have been applied to estimate human exposure to Hg and the associated risks, including dietary intake via fish consumption [41,42], water ingestion, and dermal absorption [43,44].

In recent decades, studies conducted in the Amazon have indicated Hg concentrations above the safe limit established by the World Health Organization (6.0 mg kg^−1^) in the hair of riverside populations [45]. Thus, the exposure to Hg of these people in the Amazon is a global concern [46]. The objective of this work was to evaluate human exposure to Hg in the lower Madeira River Basin (Western Amazon), a region in which previous studies have indicated relatively high Hg concentrations in human populations [23,47,48] and in fish [49,50,51]. We determined the total Hg (THg) and MeHg concentrations in the hair of residents of a riverside community of the lower Madeira River (in the Demarcação District of the municipality of Porto Velho, Rondônia State), as well as samples of surface water and the fish species most frequently consumed by the local population, aiming to answer three main questions: (i) How do gender, age, and length of time living in the community influence THg concentrations in human hair? (ii) What is the contribution of fish and water consumption to the dietary intake of THg and MeHg by the population? (iii) What is the maximum safe daily consumption of the fish species most frequently consumed by the population of the Demarcação District? In addition to supplying primary data on Hg concentrations in humans and supporting comparisons with previous studies carried out in the Amazon, especially in the Madeira River Basin, these data will enable an integrated assessment of the risks associated with Hg exposure through the consumption of fish and water in the community studied.

## 2. Materials and Methods

### 2.1. Study Area

**Figure 1:**
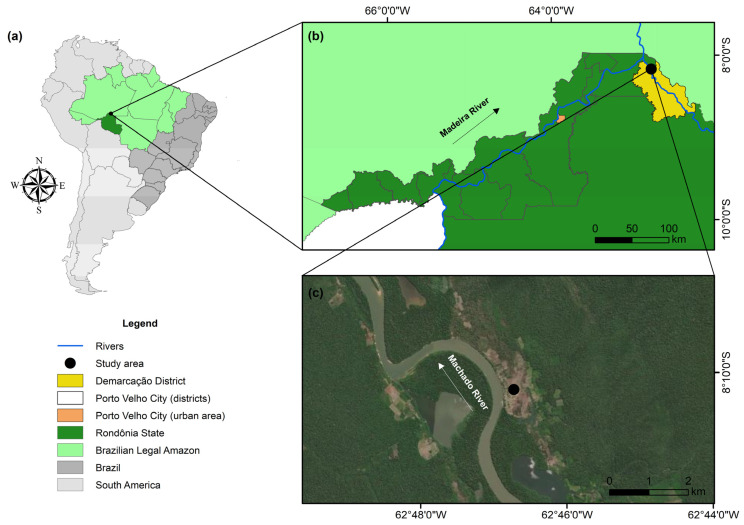
A location map of the study area representing (**a**) South America, Brazil, and the Brazilian “Legal Amazon”, (**b**) the urban area of Porto Velho, and (**c**) the Demarcação District.

Our study area was the Demarcação District in the lower Madeira River Basin in the Western Amazon (geographic coordinates: 8°10′16.20″ S and 62°46′45.30″ W; Figure 1). The Demarcação District is located on the right bank of the Machado River, 30 km from its confluence with the Madeira River and 140 km from the municipality of Porto Velho, the capital of Rondônia [52]. The Demarcação community is made up of approximately 550 inhabitants in an area of 2190 km^2^ (~4 inhabitants/km^2^) [53] whose main economic activities are fishing and subsistence agriculture, with an emphasis on cassava cultivation and the manufacture of its byproducts [54,55]. Although the Demarcação community is considered semi-isolated, with access only via the river, it is located close to the Calama District, which has greater economic development, facilitating access to products originating in urban areas [52]. The main sources of Hg in the lower Madeira River region were identified as primary emissions through gold mining and the remobilization of Hg retained in the soil through changes in land use [47,56]. Furthermore, our study area is located approximately 175 km downstream from the Santo Antônio hydroelectric plant, which presents a potential source of MeHg for fish [57]. In addition, the Demarcação District has no water or sewage treatment, and water for human consumption is collected from rivers and excavated wells (groundwater) [54]. Therefore, water consumption may be a potential route of exposure to Hg.

### 2.2. Sample Collection and Preparation

Hair samples were collected from 33 men and 32 women living in the Demarcação District (*n* = 65) in September 2022. Only adults (>18 years old) who had lived in the area for at least two years were selected. Questionnaires were applied to obtain basic data from the participants such as their age, sex, place of birth, length of residence, eating habits, protein consumption, and pregnancy or breastfeeding in women (Table 1).

Hair samples were collected in the occipital region with the aid of stainless steel scissors, approximately 1 cm above the scalp, and were stored in plastic bags at room temperature. In the laboratory, the samples were washed with a 0.01% EDTA solution (*m*/*v*, Merck) to extract exogenous materials adsorbed on the hair strands. After 24 h, the samples were washed with ultrapure water (Milli-Q Plus, Millipore, Bedford, MA, USA), dried in an oven at 40 °C, and cut with stainless steel scissors to facilitate chemical digestion. The samples were stored in collection bottles until THg and MeHg contents were determined. This study was registered with the Brazil Platform and approved by the Research Ethics Committee of the Federal University of Rondônia (CEP/UNIR, CAAE no. 57119222.0.0000.5300).

Samples of surface water (*n* = 53) and fish (*n* = 513) were also collected along the upper and lower Madeira River to calculate human exposure indices (Figure 1). Surface water samples (~10 cm) were collected in 1 L amber bottles and acidified with 4 mL of 37% hydrochloric acid (HCl) (Merck, Darmstadt, Germany). The fish were collected by local fishermen using specific nets to obtain the target species. A total of 513 specimens of fish species mentioned in the questionnaires by the residents of the Demarcação District were collected. The water and fish samples were kept under refrigeration (4 °C) and transported to the laboratory. In the laboratory, taxonomic identification was carried out, and aliquots were collected from the dorsal muscle of each fish specimen. The water samples were refrigerated and the fish samples were frozen until THg and MeHg quantification. The water and fish samples were collected in the same year as the human hair samples and were authorized by the Brazilian System for Authorization and Information on Biodiversity (SISBIO, Authorization Number 65585-6).

### 2.3. Total Mercury (THg) Determination

The chemical extraction of THg from human hair and fish samples was carried out according to Bastos et al. [58]. Approximately 50 mg of each hair sample (dry weight) and 200–400 mg of each fish sample (wet weight) were weighed according to eating habits on an analytical balance (Shimadzu AY-220, Kyoto, Japan). Subsequently, 4.0 mL of a 1:1 (*v*/*v*) solution of sulfuric acid and nitric acid (HNO_3_:H_2_SO_4_) (Merck, Darmstadt, Germany) was added, and the samples were kept in a digester block (Tecnal TE-007MP, Piracicaba, SP, Brazil) at 70 °C. After 30 min, 5.0 mL of a 5% (*m*/*v*) potassium permanganate solution (KMnO_4_) was added to oxidize the Hg, and the samples were transferred again to the digester block for 20 min. The next day, the samples were titrated by adding drops of 12% (*m*/*v*) hydroxylamine hydrochloride (NH_2_OH·HCl), and a final volume of 14 mL was completed using ultrapure water. THg quantification was performed by cold vapor generation based on atomic absorption spectrophotometry (CV-AAS, FIMS-400, Perkin-Elmer, Waltham, MA, USA) with a flow injection system (FIAS) and an automatic sampler (AS-10) operated using Winlab software (Winlab 32^TM^ for AA). The method consists of reducing Hg through a mixed solution of sodium borohydride (NaBH_4_ 0.2% *m*/*v*) and 0.05% (*m*/*v*) sodium hydroxide (NaOH), followed by oxidation with 3% (*v*/*v*) hydrochloric acid (HCl), enabling the transport of Hg^0^ to an atomic absorption cell, using argon as a carrier gas [58].

THg concentrations in unfiltered water samples were determined by oxidizing the forms of mercury [59]. Approximately 20 g of each sample was weighed in a 40 mL vial, and 100 µL of concentrated bromine monochloride (BrCl) (Brooks Rand Labs, Seattle, WA, USA), 100 µL of 30% (*w*/*v*) hydroxylamine hydrochloride (NH_2_OH·HCl) (Merck, Darmstadt, Germany) and 100 µL of 20% (*w*/*v*) stannous chloride (SnCl_2_) (Merck, Darmstadt, Germany) were added sequentially. THg concentrations were quantified by cold vapor atomic fluorescence spectrometry (CV-AFS, MERX-T, Brooks Rand, Seattle, WA, USA).

### 2.4. Methylmercury (MeHg) Determination

The MeHg concentrations in a subset of the human hair and fish samples were determined. Approximately 10 mg (dry weight) of each hair sample was weighed on a precision scale, while the fish dorsal muscle samples were wet weighed according to the species’ feeding habits: 100 mg for predator species and 200 mg (wet weight) for non-predator species. Chemical extraction was carried out by adding 3.0 mL of potassium hydroxide in a methanol solution (KOH–methanol 25%, *w*/*v*) and subsequent drying at 70 °C for 4 h, followed by homogenization in a vortex shaker (Fisatom 772, São Paulo, Brazil) every hour. After chemical extraction, the samples were stored in the dark for 48 h to avoid MeHg degradation [60]. After this period, the samples were centrifuged for 10 min at a speed of 3000 rpm (Centribio Model 80-2B, São Paulo, Brazil). The MeHg ethylation process occurred in an aqueous solution with 300 μL of 2 M anhydrous sodium acetate (NaC_2_H_3_O_2_, Sigma-Aldrich, St. Louis, MO, USA), followed by 30 μL of the sample diluted 10 times and 50 μL of 1% sodium tetraethyl borate (NaBEt_4_) (Brooks Rand Labs, Seattle, WA, USA). The final volume (40 mL) of each amber vial was completed with ultrapure water. The quantification process consisted of the derivatization of MeHg to ethylmethylmercury through packed column separation (36 °C). The chemical forms of Hg were reduced to Hg^0^ by heating the GC/pyrolysis unit, and transport to the absorption cell was carried out with ultrapure argon (99.999%), which was then carried to the atomic fluorescence detector [61].

MeHg concentrations in unfiltered water samples were determined by distillation, aqueous ethylation, purging, trapping and gas chromatography together with atomic fluorescence spectrometry [62,63]. Two hundred microliters of 1% (*w*/*v*) ammonium pyrrolidine dithiocarbamate (APDC) (Sigma-Aldrich, St. Louis, MO, USA) was added, and the samples were placed in an aluminum block (Brooks Rand Labs, Seattle, WA, USA) at 125 °C for distillation. Aliquots of the distilled samples were transferred to amber vials with a final volume of 40 mL, and 300 µL of an acetic acid buffer solution (Merck, Darmstadt, Germany) and sodium acetate (Sigma-Aldrich, St. Louis, MO, USA) were added to adjust the pH to a range of 4.5 to 4.9. The aqueous ethylation of the samples was carried out by adding 50 µL of the derivatizing agent, 1% (*w*/*v*) sodium tetraethylborate (NaBEt_4_) (Brooks Rand Labs, Seattle, WA, USA). The determination of MeHg in all matrices (fish, human hair, and water samples) was carried out using a gas chromatograph coupled to an atomic fluorescence spectrophotometer (GC-AFS) with an AFS III system (MERX-M, Brooks Rand Labs, Seattle, WA, USA) and the aid of MERX-M Guru 4.01 software.

### 2.5. Analytical Quality Control

All chemical reagents used were of high purity and obtained from Merck (Darmstadt, Germany) and/or Sigma-Aldrich (St. Louis, MO, USA), and all materials such as glassware and plastic tubes were previously sterilized in 5% (*v*/*v*) nitric acid (HNO_3_) for 48 h and rinsed with deionized water [58]. Analytical blank samples (reagents only) were used to ensure the decontamination of the glassware and the purity of the reagents used for the chemical extraction of the samples. Table 2 summarizes the limits of detection (LODs) and quantification (LOQs) of all matrices assessed, as well as the recovery rates of the spikes and certified reference materials.

### 2.6. Estimating Hg Exposure through Fish Consumption

Human exposure to Hg associated with fish consumption was evaluated by calculating the estimated daily intake (EDI) from 513 specimens of nine fish species among those most frequently consumed in the studied community. The EDI was calculated using Equation (1), where *C*_[*Hg*]_ is the THg concentration determined in the dorsal muscle of each specimen (µg kg^−1^, wet weight), *DC* is the weekly fish consumption (kg week^−1^), and *BW* is the human body weight (kg) [41]. We adopted daily fish consumption data determined in riverside communities on the Madeira River Basin which have similar characteristics to the Demarcação District (320 g day^−1^) [64]. Body weight values were obtained from a population census by the Brazilian Institute of Geography and Statistics, represented by the mean body weight values of male and female adults (18 to +65 years) living in the state of Rondônia (65 kg) [65]. The EDI results should be interpreted according to two major assumptions: (i) the cooking method did not cause significant changes in THg concentrations in the fish [66,67]; and (ii) since the fish species from the Madeira River Basin had high mean MeHg:THg ratios (70–100%) [50,51]. THg was used as a proxy for the MeHg concentration.

(1) $$Estimated\;Daily\;Intake\;(EDI)=\frac{C_{\left[Hg\right]\times }\;DC}{BW(\mathrm{kg})}$$

The risks associated with dietary exposure to Hg were estimated by comparing the EDI with the Provisional Tolerable Weekly Intake (PTWI) recommended by the Joint FAO/WHO Expert Committee on Food Additives (JECFA) [68]. The PTWI represents the maximum amount of MeHg in fish, expressed in body weight per week, that can be ingested weekly without significant risks to human health (1.6 μg kg^−1^) [68]. Considering that the participants in our study consumed fish two to six times a week (Table 1), we divided the PTWI value by seven to obtain the recommended daily limit (PTWI/7 = 0.23 μg kg^−1^ day^−1^). In addition, the risk ratio (RR) was also used to estimate the risk to human health, where *EDI_Abs_* is the fraction of Hg absorbed by the human body through fish consumption (80% of EDI) and *RfD* represents the daily oral chronic reference dose of MeHg (0.1 μg kg^−1^) [69] (Equation (2)) [27]. RR values greater than or equal to one indicate that the dose of MeHg absorbed by a test subject is higher than the reference dose.

(2) $$Risk\;Ratio\left(RR\right)=\frac{{EDI}_{Abs}}{RfD}$$

The maximum safe consumption quantity (MSCQ) for the fish species consumed by the community (g day^−1^) was calculated using Equation (3) [42], where *BW* is the average body weight of the population (65 kg), *RfD* is the reference dose of MeHg (0.0001 mg kg^−1^), and *C*_[*Hg*]_ is the THg concentration determined in the fish (µg kg^−1^, wet weight).

(3) $$Maximum\;Safe\;Consumption\;Quantity\;\left(MSCQ\right)=\frac{BW\times RfD}{C_{\left[Hg\right]}}\times 1\times 10^{3}$$

### 2.7. Estimating Hg Exposure through Water Ingestion

Human exposure to THg and MeHg via unfiltered water was estimated through the average daily dose (ADD) ingested, measured in mg kg^−1^ per day. The ADD values were calculated using Equation (4) (adapted from the U.S. EPA [62] and Mestanza-Ramón et al. [44]), where *C*_[*Hg*]_ is the mean concentration of THg or MeHg in the water (mg L^−1^), *IR* is the ingestion rate (2 L day^−1^), *CF* is the conversion factor (1 × 10^−6^ kg mg^−1^)*, EF* is the frequency of exposure (365 days per year), and *ED* is the duration of exposure, obtained from the third quartile of the distribution of residence time values in the Demarcação District (24 years). Our local *ED* value is in accordance with the value recommended by the United States Environmental Protection Agency (U.S. EPA) and applied in other recent studies (30 years) [43,44,70]. *BW* is the average body weight, previously established as 65 kg [65]. *AT* is the exposure period for non-carcinogenic effects, calculated by multiplying the *ED* and *EF* (8760 days).

(4) $$Average\;Daily\;Dose\;\left(ADD\right)=\frac{C_{\left[Hg\right]}\times EF\times IR\times ED\times CF}{AT\times BW}$$

The hazard quotient (HQ) was used to assess the non-carcinogenic risk associated with water ingestion, calculated using the ratio between the ADD and the daily oral chronic reference dose of Hg (*RfD*) (Equation (5)) [43]. The *RfD* is 0.0004 mg kg^−1^ for THg (IHg and MeHg) and 0.0001 mg kg^−1^ for MeHg [69].

(5) $$Hazard\;Quotient\left(HQ\right)=\frac{ADD}{RfD}$$

### 2.8. Data Analysis

Descriptive statistics for the THg and MeHg concentrations were calculated, represented by the mean, standard deviation, and range. MeHg quantification was performed on hair samples from only 22 participants due to the small mass of the samples. Therefore, only THg concentrations were included in the inferential statistical analyses. The Kolmogorov–Smirnov (*n* > 50) and Shapiro–Wilk (*n* < 50) tests were used to evaluate data distribution. An analysis of covariance (ANCOVA) was used to compare THg concentrations between male and female individuals (mean least square–MLS), including age and length of residence as quantitative covariates. Additionally, we grouped male and female individuals into three age classes: young (18–24 years old), adult (25–64 years old), and elderly (+65 years old). The difference in THg concentrations between age classes was assessed using an analysis of variance (ANOVA) and Tukey’s post hoc test.

The data were transformed using the Box–Cox technique to meet the assumptions of the parametric tests. The homogeneity of variance of the residuals was assessed using Levene’s test, and the normality of the residuals was assessed using the Kolmogorov–Smirnov (ANCOVA) and Shapiro–Wilk (ANOVA) tests. The significance level adopted for all analyses was α = 0.05. The graphical representation of the results was performed with untransformed data. A post hoc assessment of the power of the analysis was performed, considering the probability of detecting large effect sizes (Cohen’s f = 0.40), aiming to avoid type I and II errors. All statistical tests showed a satisfactory analysis power (between 0.8 and 1). Statistical analyses were performed using XLSTAT Premium 2023.2 software, and the power of the analyses was evaluated using the free software GPower 3.1.

## 3. Results

### 3.1. Effects of Gender and Age on THg Concentrations in Human Hair

**Figure 2:**
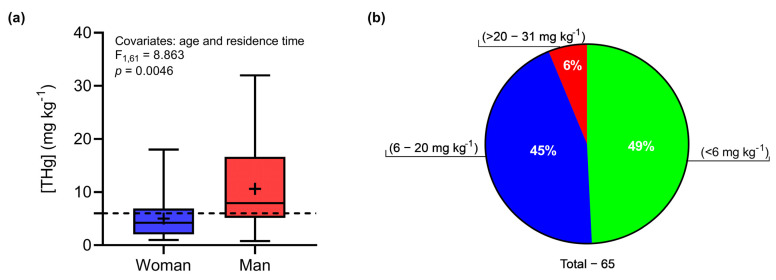
(**a**) Difference in THg concentrations between women and men. The dashed line indicates the safe limit recommended by the WHO. The central line represents the medians and horizontal limits of the rectangles, which are the first and third quartiles. The dashes at the tips of the vertical lines represent the maximum and minimum values, and the average is represented by +. (**b**) Proportions of THg concentrations in all individuals evaluated.

The average THg concentration in the hair of residents of Demarcação was 7.86 ± 6.78 mg kg^−1^ (0.78–31.97 mg kg^−1^). An analysis of covariance (ANCOVA) indicated a significant effect of gender (LSM: F_1,61_ = 8.863; *p* = 0.0046) on THg concentrations in hair. Women had a mean THg concentration equal to 5.01 ± 3.89 mg kg^−1^ (0.97–18.02 mg kg^−1^), while the mean concentration determined in men was 10.62 ± 7.83 mg kg^−1^ (0.79–31.97 mg kg^−1^) (Figure 2a). Figure 2b represents the proportions of the THg concentrations in the Demarcação population in relation to limits established by regulatory agencies. Forty-nine percent of individuals (*n* = 32) had THg concentrations below that established by the WHO (6.00 mg kg^−1^) [71], with a low probability of harmful effects on the population. However, 44% of individuals presented concentrations above the safe limit, with concentrations close to 20.0 mg kg^−1^ (*n* = 29), which could be harmful to human health if not properly controlled. Only 6% of individuals had concentrations above 20.00 mg kg^−1^ (*n* = 4). The average MeHg concentration was 5.52 ± 5.42 mg kg^−1^ (0.40–20.10 mg kg^−1^), with an average MeHg:THg ratio equal to 67 ± 22% (20–96%, *n* = 22; 19 women and 3 men).

**Figure 3:**
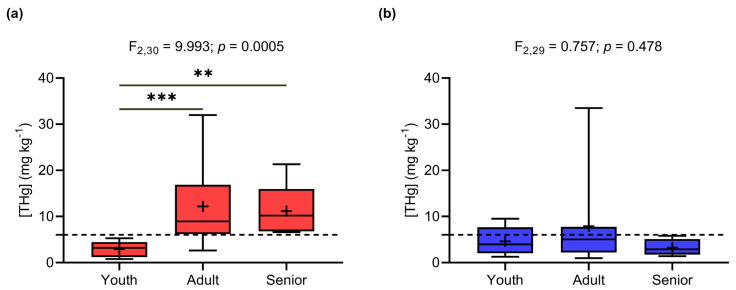
THg concentrations in different age classes in (**a**) men and (**b**) women (** *p* < 0.005; *** *p* < 0.0005). The dashed line indicates the safe limit recommended by the WHO (6.0 mg kg^−1^).

Age had a significant and positive effect in relation to THg concentrations in males (LSM: F_1,61_ = 4.147; *p* = 0.046). Time of residence in the Demarcação District did not influence THg concentrations (*p* > 0.05). Considering the ANCOVA results, individuals of both genders were grouped into age classes for a more comprehensive comparison. An analysis of variance (ANOVA) indicated a significant difference in THg concentrations between age classes in males (ANOVA: F_2,30_ = 9.993; *p* = 0.0005; Figure 3a), according to which adults (12.19 ± 8.12 mg kg^−1^) and elderly people (11.14 ± 5.97 mg kg^−1^) had significantly higher THg concentrations than young people (2.90 ± 1.75 mg kg^−1^, Tukey: *p* = 0.0004 and 0.0044, respectively). There was no significant difference between female age groups (*p* < 0.05), with average concentrations of 4.61 ± 3.03 mg kg^−1^ in young women, 7.89 ± 8.84 mg kg^−1^ in adults, and 3.52 ± 1.82 mg kg^−1^ in elderly women (Figure 3b).

### 3.2. THg Concentrations in Fish Species Consumed by the Population

Based on the results of a questionnaire, freshwater fish species were the main source of protein for the population of Demarcação. No marine fish species were reported. The fish consumed were either bought at a local market or caught by hand by the people themselves. Most of the fish species consumed had herbivorous (40%) and detritivorous (35%) feeding habits, followed by carnivorous, (10.5%), omnivorous (10.5%), piscivorous (3%), and planktivorous (1%) feeding habits. Among the eighteen species identified, the three species most frequently consumed were *Mylossoma* spp. (37%) *Semaprochilodus* sp. (24%), and *Pseudoplatystoma punctifer* (8%). 

Table 3 presents the mean THg concentrations in the main fish species most frequently consumed by riverside communities of the lower Madeira River Basin. Among the samples evaluated in our study, only 4% of the fish specimens (*n* = 19) exceeded the limit of Brazilian legislation for non-predator fish (0.50 mg kg^−1^) and predator fish (1.00 mg kg^−1^) [72], represented by the following species: *Cichla* sp. (*n* = 10), *Triportheus* spp. (*n* = 4), *Schizodon fasciatus* (*n* = 2), *Hypophthalmus marginatus* (*n* = 2), and *Mylossoma* sp. (*n* = 1).

MeHg concentrations were quantified in 14 fish specimens that had THg concentrations above the safe limits for predator (*n* = 7) and non-predator species (*n* = 7). The mean MeHg concentration was 0.98 ± 0.46 mg kg^−1^, ranging from 0.40 to 1.70 mg kg^−1^. The MeHg:THg ratio was 98 ± 46%, with no significant difference between predator (91–104%) and non-predator species (59–105%). These results reinforce the suitability of determining THg as a proxy for the MeHg level in freshwater fish. MeHg:Hg ratios above 100% (up to 120%) were considered acceptable due to the different analytical methods and the sensitivity of the equipment used to calculate THg and MeHg concentrations.

### 3.3. Human Exposure through Fish Intake and Maximum Safe Consumption Quantity

**Figure 4:**
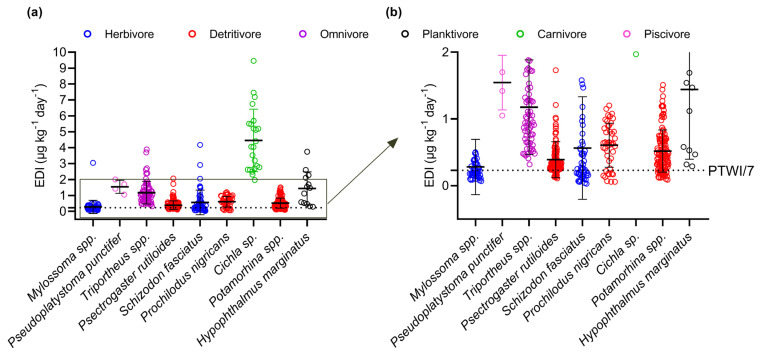
Estimated daily intake (EDI) in nine species among the species most mentioned by the population of the Demarcação District (*n* = 513 specimens). Graph (**a**) represents all the EDI values, while graph (**b**) is a cutout of the EDI values to better visualize their distribution in relation to the PTWI (PTWI/7 = 0.23 μg kg^−1^), indicated by the dashed line. Different colors are used to differentiate the species by feeding habits.

Human exposure through fish consumption was assessed for nine species collected in our study (Figure 4, Table 4). Considering all the species evaluated, the estimated ingestion rate showed broad variability, with a mean daily ingestion of 0.77 ± 1.06 μg kg^−1^, approximately 3.3 times higher than the PTWI/7 (0.23 μg g^−1^). According to the questionnaires, residents of the community studied consume fish between two and six days a week, representing estimated weekly intakes between 1.54 and 4.62 μg kg^−1^. Therefore, the average weekly intake estimates in the community studied vary from 96 to 336% in relation to the PTWI (1.60 μg kg^−1^).

The dietary intake of 79% of the specimens resulted in Hg exposure higher than the PTWI (*n* = 408) (Figure 4). All specimens of the species *Cichla* sp. (4.46 ± 1.96 μg kg^−1^), *Pseudoplatystoma punctifer* (1.54 ± 0.41 μg kg^−1^), *H. marginatus* (1.44 ± 1.04 μg kg^−1^), and *Triportheus* spp. (1.18 ± 0.71 μg kg^−1^) exceeded the PTWI. In contrast, the highest proportions of specimens with an estimated intake below the PTWI were represented by the herbivores *Mylossoma* spp. (0.28 ± 0.41 μg kg^−1^) and *S. fasciatus* (0.56 ± 0.77 μg kg^−1^), followed by the detritivores *Potamorhina* spp. (0.52 ± 0.31 μg kg^−1^), *P. nigricans* (0.61 ± 0.33 μg kg^−1^), and *Psectrogaster rutiloides* (0.39 ± 0.27 μg kg^−1^). It is important to note that considering our entire sample population of *Mylossoma* spp. (*n* = 50), the species most mentioned by the population, the EDI values of 32 specimens were above the recommended level (64%). Regarding the risk ratio, 95% of the samples exceeded the cutoff value (RR ≥ 1, *n* = 487). Only individuals belonging to herbivorous and detritivorous species had RR values < 1, namely, *S. fasciatus* (*n* = 10), *Mylossoma* spp. (*n* = 8), *Potamorhina* spp. (*n* = 4), *P. nigricans* (*n* = 3), and *P. rutiloides* (*n* = 1).

The maximum safe consumption quantity (MSCQ) of our entire species pool was 99 ± 96 g day^−1^ (3–929 g day^−1^), approximately three times lower than the average daily consumption of riverine populations in the lower Madeira River Basin (320 g day^−1^). The lowest MSCQ values were determined for the predator species *Cichla* sp. (9 ± 4 g day^−1^) and *P. puntictifer* (22 ± 6 g day^−1^), while the herbivorous species *S. fasciatus* had the highest average MSCQ and the highest proportion of individuals with MSCQ values above the average daily consumption (16%, 174 ± 186 g day^−1^). For *Mylossoma* spp., only four individuals had an MSCQ greater than 320 g (8%), and the maximum recommended consumption was 175 ± 93 g day^−1^. Complete descriptive statistics for the EDI and MSCQ values are shown in Table 4.

### 3.4. Human Exposure through Water Ingestion 

Table 5 shows the concentrations of THg and MeHg and the MeHg:THg ratio in water, as well as daily exposure and associated risks. The mean THg and MeHg concentrations in the unfiltered water samples were 7.04 ± 6.42 ng L^−1^ and 0.15 ± 0.11 ng L^−1^, respectively (*n* = 53). The average MeHg:THg ratio was relatively low (4 ± 5%) and had high variability, ranging from 0.5 to 24.5%. The mean estimated daily intakes of THg and MeHg were 1.66 × 10^−2^ ± 1.52 × 10^−2^ µg kg^−1^ and 3.65 × 10^−4^ ± 2.52 × 10^−4^, respectively. The HQ results indicated low probabilities of non-carcinogenic risks from water ingestion, with values substantially below 1 in all samples (Table 5).

## 4. Discussion

### 4.1. Comparison of THg Concentrations in Human Hair with Other Amazonian Communities

The hair THg concentrations determined in our study can be compared with those reported in other communities in the Amazon, including in Rondônia State (Table 6). One of them is the population residing around the large Balbina Reservoir (Amazonas State) [73], where the damming and alteration of the natural flow of the river and the depth of the reservoir ended up reducing the available oxygen, leaving the aquatic environment anoxic and with high sedimentation. Thus, the microorganisms present in the bottom sediment transfer a methyl group (-CH_3_) from the decomposed matter to Hg^2+^. From there, the methylation process by sulfate-reducing bacteria begins and, consequently, levels of MeHg concentrations increase [5,12,15].

Our results are also within the range of THg concentrations determined in communities impacted by ASGM. Indeed, the proportion of individuals with hair THg concentrations above the safe limit recommended by the WHO (6.0 mg kg^−1^) [74] was very similar between our study (51%) and a study carried out in native communities impacted by ASGM in the Peruvian Amazon (60%) [74]. The same pattern was observed in indigenous and riverside communities in the Tapajós River Basin [75,76]. It is noteworthy that the communities evaluated by Basta et al. [76] have an average weekly consumption of fish similar to that determined in our study (three times a week) and consume species mentioned by the population of the Demarcação District, reinforcing the importance of fish consumption for human exposure to Hg.

The THg concentrations observed in our study were substantially lower than those observed in Demarcação between 2001 and 2003 (W. R. Bastos, unpublished data). This result is contrary to that determined in a riverine population living around the Tucuruí Reservoir (Amazonas), in which no significant differences in Hg concentrations were observed in the population over a 20-year interval [77,78]. The population evaluated by Arrifano et al. [78] is isolated and underwent few economic changes in the time interval between the two studies, so there was no access to electricity, and fish represented the main source of protein. Therefore, the temporal reduction determined in our study reflects two main changes that occurred in the riverside populations of the Madeira River Basin: (i) a reduction in gold mining activities; and (ii) changes in the living conditions of the riverside residents due to the federal government’s social programs. The gold mining cycle in the Madeira River reached its peak between 1970 and 1980 and significantly decreased from the 1990s onward [79]. Furthermore, new technologies have been adopted in recent decades to reduce Hg emissions during the amalgamation process, such as distillers (crucibles or “retorta”, as they are called by miners). Thus, despite the remobilization of Hg due to changes in land use and occupation [56], the temporal difference in Hg concentrations in the Demarcação population can be attributed to a reduction in primary Hg emissions in recent decades.

Additionally, there have been major changes in the economic patterns of riverside populations in the Brazilian Amazon since the 2000s, including populations in the Madeira River Basin [80]. These changes have been driven by federal government initiatives providing financial subsidies to households in situations of social vulnerability (the “Bolsa Família” program) [81,82] and promoting electrification in rural and remote regions of the Amazon (the “Luz para Todos” program) [83]. Access to electricity, the diversification of forms of income generation, and the acquisition of motorized boats has enabled access and adequate storage conditions for ultra-processed foods as an alternative to fish consumption (nutritional transition) [84,85,86]. However, there is a tradeoff between changes in eating habits and Hg accumulation. Despite the reduction in direct exposure through fish consumption, the intake of ultra-processed foods results in high levels of cholesterol and triglycerides and the accumulation of body fat (overweight) [87,88]. Thus, considering that MeHg has a lipophilic character, excess body fat can result in greater potential for bioaccumulation in the human body, even with a reduction in fish consumption [80].

**Table 6:** Summary of THg concentrations (mg kg^−1^, mean ± standard deviation, minimum–maximum, and *n*) in human hair from riverside populations in the Amazon. The asterisk (*) next to the reference indicates that significant differences were found in THg concentrations between genders.

Location	*N* (Adults)	General	Male	Female	References
Demarcação (RO) Madeira River	65	7.87 ± 6.78 (0.78–31.97)	10.62 ± 7.83 (0.79–31.97)	5.01 ± 3.89 (0.97–18.02)	This study *
Demarcação (RO) Madeira River	4	28.01 ± 7.35 (22.07–37.07)	–	–	W. R. Bastos (unpublished data)
Calama (RO) Madeira River	24 a	9.23 ± 5.78 (1.05–22.48)	9.61 ± 3.24 (4.34–14.53)	8.76 ± 6.37 (1.05–22.48)	[47]
Cujubim (RO) Madeira River	12 a	6.30 ± 4.00 (1.55–14.67)	7.22 ± 5.18 (1.56–14.67)	6.76 ± 4.36 (1.55–14.67)	[47]
Firmeza (RO) Madeira River	3 a	11.80 ± 2.75 (9.40–14.80)	12.10 ± 3.89 (9.40–14.80)	11.20	[47]
Itacoã (RO) Madeira River	6 a	11.97 ± 4.33 (5.28–16.00)	11.51 ± 5.57 (5.28–16.00)	12.42 ± 3.91 (7.90–14.76)	[47]
Nazaré (RO) Madeira River	27 a	12.14 ± 6.34 (1.08–22.60)	11.91 ± 7.48 (2.48–22.46)	11.65 ± 5.95 (1.08–22.60)	[47]
Papagaios (RO) Madeira River	8 a	12.73 ± 7.37 (4.76–27.22)	16.14 ± 7.37 (6.66–27.22)	9.90 ± 4.84 (4.76–17.20)	[47]
Santa Rosa (RO) Madeira River	6 a	13.30 ± 2.54 (8.56–15.44)	14.88 ± 0.81 (13.95–15.44)	11.73 ± 2.84 (8.56–14.08)	[47]
São Carlos (RO) Madeira River	14 a	8.71 ± 5.77 (1.84–22.83)	8.94 ± 5.48 (3.30–17.69)	8.59 ± 6.24 (1.84–22.83)	[47]
Terra Caída (RO) Madeira River	5 a	10.65 ± 3.61 (5.01–14.61)	-	14.61	[47]
Puruzinho Lake (AM) Madeira River	4 a	20.71 ± 5.10 (14.21–28.27)	20.22 ± 7.25 (14.21–28.27)	22.18	[47]
Barreiras (PA) Tapajós River	89 a	13.74 ± 4.52 (2.07–20.87)	13.42 ± 5.12 (2.07–20.87)	14.03 ± 4.16 (7.16–20.39)	[75]
Sawré Muybu Indigenous Land (PA) Tapajós River	116 a	8.3 (2.0–22.8)	8.8 ± 4.6 (2.6–22.8)	7.8 ± 3.8 (2.0–20.2)	[76]
Balbina Village (AM) Uatumã River	25 a	6.4 1.2–15.5	5.5 ± 3.5 (1.2–12.2)	7.4 ± 4.6 (2.2–15.5)	[73]
Tucuruí Hydropower plant (PA) Tocantins River	108	-	11.5 ± 11.8	8.8 ± 8.0	[89] *
Tocantins River (PA)	37	12.0 (7.9–23.8) b (1.1–75.8)	19.7 (6.2–47.6) b (1.1–75.8)	11 (8–18) b (3.6–42.9)	[81]

(a) sample size and general THg concentration considering only adult individuals. (b) median (interquartile range).

### 4.2. Effects of Gender and Age on THg Concentrations in Human Hair 

Our results indicated a significant difference in THg concentrations between genders, with higher concentrations in the hair of males. There is no consensus in the literature about the disparity in THg concentrations between men and women, so significant differences are often not detected [25,75,78,90]. However, patterns similar to those in our study were determined in studies conducted among populations living in the Brazilian Amazon [24,89] and in neighboring regions of South America [91]. Although there was no significant difference in the frequency of fish consumption between genders in our study, there was a generally greater consumption of fish per meal by men compared to women (g meal^−1^), increasing exposure to Hg [24,78,91]. In addition, the difference between genders can be affected by confounding factors such as the mobilization of Hg absorbed in the hair through treatments frequently carried out by women (e.g., the use of a hairdryers and dyes) [92] and women’s natural physiological elimination pathways (e.g., urine, breast milk, and the placenta) [93]. Indeed, high THg concentrations were determined in the milk of postpartum women living along the Madeira River, including the Demarcação District (0.12–6.47 mg kg^−1^) [38], resulting in high THg concentrations in the hair of children in this region [52]. However, considering that only two pregnant women and one lactating woman were included in our sample, the difference in THg concentration related to sex is mainly attributed to the greater intake of fish by men.

According to the questionnaire responses, the main source of exposure to Hg is through the consumption of freshwater fish. No participants of either gender work in ASGM or have been exposed to other potential sources of Hg. Therefore, considering that few studies have determined significant differences in the pattern of Hg accumulation between genders, our data add new evidence of this pattern in Amazonian riverside communities. Our main hypothesis for the difference in Hg accumulation related to gender is the higher consumption of fish per meal (g meal^−1^) by males.

We also identified a positive effect of age on Hg concentrations in males, with significantly higher concentrations in adults (25–64 years) and elderly people (>64 years) compared to young people (18–24 years). These results are in line with previous studies conducted with riverside populations in the Amazon [22,94]. Furthermore, the relationship between Hg in hair and age was also significant only for males in communities in Cambodia, Southeast Asia [95]. In contrast, studies have indicated that younger individuals have higher metabolic rates, resulting in greater food consumption and consequently higher Hg concentrations in the body [96]. However, as mentioned above, the opposite pattern found in our study may be associated with the nutritional transition observed in riverside populations in the Amazon. Considering that the federal government’s social programs (“Bolsa Família” and “Luz para Todos”) were implemented in the early 2000s, the youngest individuals evaluated in our study were raised on more diversified diets, reducing exposure from fish consumption. On the other hand, the higher Hg concentrations observed in older individuals are closely associated with the bioaccumulation process in the body, since individuals who eat fish with high concentrations for prolonged periods have higher levels of chronic exposure to Hg [24]. This is in line with a study carried out in Wisconsin (U.S.A.), where older individuals ate fish more frequently and had higher Hg concentrations compared to younger individuals [92]. Additionally, the faster metabolism of young people implies higher rates of Hg excretion through urine [97], reducing its bioaccumulation in the body.

### 4.3. Human Exposure through Diet and Water

The results of our questionnaire indicated that the main fish species consumed by the Demarcação population are herbivores and detritivores (75%), with an emphasis on *Mylossoma* spp. (Pacú) and *Semaprochilodus* sp. (Jaraqui). According to our results and a large-scale assessment of Hg concentrations in fish from the Madeira River Basin, individuals of the genera *Mylossoma* spp. and *Semaprochilodus* sp. presented relatively low THg concentrations, mostly below the safe limits established by Brazilian and international regulatory agencies (Table 3) [50]. Fish that occupy higher trophic levels (e.g., carnivores and piscivores) have higher rates of MeHg bioaccumulation in their tissues, while species at the base of the food chain (e.g., herbivores and detritivores) have lower concentrations [98,99]. This pattern was reflected in our results, in which all individuals of species with a predatory or generalist feeding habit had daily intake estimates above the PTWI. 

Among predatory species, *Cichla* sp. stands out, with a daily intake 8 to 41 times higher than the PTWI. The highest MSCQ recommended for *Cichla* sp. was 16 g per day, 20 times lower than the consumption determined in riverine populations in the Madeira River Basin (320 g per day) [64], which was used in our study as a reference for calculating daily intake. Species of the *Cichla* genus are voracious predators and obtain food at different trophic levels during their ontogenetic development [100], increasing the potential for Hg bioaccumulation. In addition, a 35-year assessment in the Madeira River Basin indicated a significant temporal increase in Hg concentrations in the species *Cichla pleiozona*, attributed mainly to anthropogenic changes in land use [101]. In contrast, despite greatly exceeding the MSCQ, herbivorous and detritivorous species had the highest proportions of species with estimated intakes below the PTWI and a lower probability of risk to human health (RR < 1). According to Lacerda et al. [102], Hg concentrations in the detritivorous species *Prochilodus nigricans* have remained consistently below safe limits over the last three decades. Thus, although fish represent an important source of protein for Amazon riverine populations, the greater frequency of consumption of non-predatory fish species can buffer the accumulation of THg in these populations [22]. 

Along these lines, recent studies have indicated that fish species that occupy low trophic levels have higher concentrations of selenium (Se) compared to predator species [25,103]. The strong association between Hg and Se favors the formation of insoluble complexes, reducing the bioavailability and toxicity of Hg [67,104]. 

Although the dietary intakes of herbivorous and detritivorous species were below the PTWI, the consumption of 79% of the specimens collected exceeded the PTWI. This result is consistent with other assessments carried out in the Amazon, where intake estimates far exceeded the recommended safe limits [76,99,102,105]. This is mainly due to the high quantity of fish consumed by riverside communities, with average values frequently above 300 g day^−1^ [22,64,106]. In the neighboring Lake Puruzinho area, the 2.5 times higher consumption frequency of detritivorous species compared to omnivorous species resulted in significantly higher daily intake estimates for detritivorous fish, regardless of lower Hg concentrations [107]. Thus, it is important to note that even with relatively low Hg concentrations in species occupying lower trophic levels, a high frequency of consumption can pose risks to human health. 

The mean Hg concentration in the water was in accordance with values determined in the Madeira River Basin between 2010 and 2018 [108], but it was 2.5 to 5 times lower than those ascertained in the region in the early 1990s [107], a period marked by intense Hg emissions from ASGM activities. The concentrations determined in our study are significantly lower than those found in areas impacted by ASGM in the Ecuadorian Amazon (500–11,220 ng L^−1^) [43,44]. In our study area, human exposure to Hg through water was negligible compared to fish consumption. Indeed, none of the samples exceeded the limit established by Brazilian legislation (200 ng L^−1^) [109]. In addition, the MeHg:THg ratios were relatively low, reducing the absorption of Hg in the human body. Therefore, fish consumption is the main route of dietary exposure to Hg.

## 5. Strengths and Limitations

We evaluated human exposure to Hg in the riverside population of the Demarcação District, located along the lower Madeira River (Western Amazon), aiming to understand Hg dynamics in populations far from urban centers and whose main protein source is freshwater fish (a source of THg and MeHg). In addition to comparing our findings with past data in the region, our results indicated different patterns of THg accumulation between genders and age groups. Considering that few studies have observed significant differences in the pattern of Hg accumulation between genders, our data add new evidence of this pattern in Amazonian riverside communities. Notably, the quantification of THg in water and fish samples supported an integrated assessment of daily intake estimates and risks associated with Hg exposure through diet. However, it is important to note some limitations of our study. (i) THg concentrations in human hair were determined in a relatively small number of samples (*n* = 65), resulting in high heterogeneity in the characteristics of our sample population that could influence Hg accumulation patterns, such as age and length of residence in the study area. (ii) There was an imbalance in the number of samples between the age classes due to the wider age range of adults (25–64 years) compared to youths (18–24 years) and seniors (+65 years). (iii) Although THg concentrations were determined in all participants, MeHg concentrations were quantified in only 22 participants due to insufficient sample mass. Hence, it was not possible to make statistical inferences about MeHg concentrations; only a descriptive analysis of the data was possible. Future studies in the Madeira River basin should consider a more representative sample size and focus on the quantification of MeHg in human hair to provide more accurate information on the influence of fish consumption on human exposure.

## 6. Conclusions

THg concentrations in the human hair of the Demarcação population were within the range reported for communities with high rates of fish consumption and which are potentially impacted by disordered land use and ASGM. THg concentrations in human hair were significantly higher in men, with an increasing trend related to age. There was no significant difference between female age groups. Time of residence in Demarcação also did not influence THg concentrations. Considering the subset of samples used for MeHg quantification, the mean MeHg:THg ratio indicates the prevalence of Hg in its organic chemical form.

The risk indices indicated low probabilities of non-carcinogenic risks from water ingestion. Indeed, human exposure to Hg through water was negligible compared to fish consumption. Most of the fish species consumed have herbivorous or detritivorous feeding habits, which showed higher proportions of specimens with daily intake and associated risk below the safe limits. However, considering all the species evaluated, the estimated ingestion rate showed broad variability, with a mean ingestion of 0.77 ± 1.06 μg kg^−1^ per day. The average weekly intake estimates in the community studied varies between 1.54 and 4.62 μg kg^−1^ (2 to 6 days). The dietary intake of 79% of the specimens had higher Hg exposure than the PTWI. Hence, the consumption of 95% of the specimens evaluated exceeded the risk ratio cutoff value (RR ≥ 1). Therefore, despite the fact that in general, Hg concentrations in fish are in line with the safe limit established by Brazilian legislation, a high frequency of consumption can pose risks to human health. 

The MSCQ for our entire species pool was 99 ± 96 g day^−1^ (3–929 g day^−1^), approximately three times lower than the average daily consumption by riverine populations in the Madeira River Basin. The species with the highest safe daily consumption quantities were herbivores and detritivores, while carnivorous species can pose potential risks to human health even if consumed in low quantities. For *Mylossoma* spp., the species most mentioned by the population, the maximum recommended consumption was 175 ± 93 g day^−1^. Thus, our results provide updated information on the level of Hg exposure in a riverside population of the lower Madeira River Basin, a region that has undergone major changes with respect to the population’s dietary habits over the last two decades. This information provides an overview of the pathways of dietary Hg intake, which is essential for promoting public policies and supporting decision making on a local and regional scale given that the Demarcação District is a riverside community representative of the Amazon. 

## Figures and Tables

**Table 1:** A summary of the characteristics of the population of the Demarcação District (Rondônia, Brazil). Values are represented by the number of individuals (% frequency) or the mean ± standard deviation (minimum–maximum).

	Total	Men	Women
Gender	65 (100%)	33 (51%)	32 (49%)
Age (years)	41.5 ± 17.6 (18–84)	42.3 ± 18.5 (19–79)	40.6 ± 16.8 (18–84)
Age Classes			
Young	13 (20%)	5 (15%)	8 (25%)
Adult	43 (66%)	23 (70%)	20 (62.5%)
Elderly	9 (14%)	5 (15%)	4 (12.5%)
Residence Time (years)	19.01 ± 16.1 (2–71)	19.7 ± 17.7 (2–71)	18.2 ± 14.6 (2–56)
Fish Consumption (meals per week)	3.6 ± 0.7 (2–6)	3.5 ± 0.6 (2–4)	3.75 ± 0.7 (3–6)
Pregnant	-	-	2 (6%)
Lactating	-	-	1 (3%)

**Table 2:** LOD, LOQ, and accuracy of analytical methods. All concentrations are represented in mg kg^−1^ except water (ng L^−1^).

Matrices	LOD	LOQ	Reference Material	Recovery (%)
THg				
Hair	0.0008	0.0024	BCR-463	119
Fish	0.0085	0.0255	DORM-2	100
Water	0.0002	0.0006	Spikes	104
MeHg				
Hair	0.004	0.012	BCR-463	108
Fish	0.000016	0.000048	DORM-2	96
Water	0.000014	0.000042	Spikes	95

BCR-463 (tuna fish: THg, 2.85 ± 0.16 mg kg^−1^; MeHg, 3.04 ± 0.16 mg kg^−1^). DORM-2 (dogfish muscle: THg, 4.64 ± 0.26 mg kg^−1^; MeHg, 4.47 ± 0.32 mg kg^−1^).

**Table 3:** Summary of THg concentrations (mg kg^−1^, mean ± standard deviation, minimum–maximum, and *n*) in the main fish species with the highest consumption by the riverside population of this study, indicating the name (scientific and common), eating habits and frequency (%) of each species mentioned in the questionnaire.

Scientific Name (Common Name)	Frequency (%)	Feeding Habit	THg (mg kg^−1^)			Reference
			Mean ± SD	Min–Max	*N*	
*Mylossoma* spp. (Pacú)	37	Herbivore	0.06 ± 0.08	0.02–**0.82**	50	This study
*Semaprochilodus* sp. (Jaraqui)	24	Detritivore	0.17 ± 0.05	0.10–0.24	8	[50]
*Pseudoplatystoma puntictifer* (Pintado)	8	Carnivore	0.31 ± 0.08	0.21–0.41	4	This study
*Triportheus* spp. (Sardinha)	7	Omnivore	0.24 ± 0.14	0.06–**0.79**	70	This study
*Psectrogaster rutiloides* (Branquinha)	4	Detritivore	0.08 ± 0.05	0.02–0.42	135	This study
*Prochilodus nigricans* (Curimatá)	3	Detritivore	0.12 ± 0.07	0.01–0.24	44	This study
*Schizodon fasciatum* (Piau)	3	Herbivore	0.11 ± 0.16	0.01–**0.85**	51	This study
*Hemiodus microlepis* (Jatuarana)	2.5	Detritivore	0.08 ± 0.09	0.01–**0.53**	61	[50]
*Cichla* spp. (Tucunaré)	2.5	Carnivore	0.91 ± 0.40	0.40–**1.92**	23	This study
*Geophagus* spp. (Cará)	1.5	Omnivore	0.14 ± 0.07	0.06–0.23	8	[50]
*Pseudoplatismtoma tigrinum* (Surubim)	1.5	Piscivore	0.48 ± 0.23	0.24–**1.20**	16	[50]
*Brachyplatystoma rousseauxii* (Dourada)	1	Piscivore	0.89 ± 0.89	0.01–**6.54**	96	[50]
*Piaractus brachypomus* (Pirapitinga)	1	Omnivore	0.07 ± 0.05	0.01–0.22	20	[50]
*Colossoma macropomum* (Tambaqui)	1	Omnivore	0.15 ± 0.13	0.02–**0.55**	42	[50]
*Potamorhina* spp. (Chora)	1	Detritivore	0.11 ± 0.06	0.02–0.31	123	This study
*Brachyplastystoma filamentosum* (Piraíba)	1	Piscivore	**1.48** ± 0.88	0.28–**3.80**	30	[50]
*Hypophthalmus marginatus* (Mapará)	1	Planktivore	0.29 ± 0.21	0.06–**0.76**	13	This study

**In Bold:** THg concentrations that exceeded the limits of Brazilian legislation.

**Table 4:** Descriptive statistics for the estimated daily intake (EDI, μg kg^−1^), risk ratio (RR), and maximum safe consumption quantity (MSCQ, g day^−1^) (mean ± standard deviation, minimum–maximum, and *n*). The proportion (%) of samples of each species that showed EDI > PTWI/7, MSCQ < the population’s daily consumption, and RR ≥ 1 are also represented in the table.

Scientific Name (Common Name)	*N*	EDI	>PTWI/7	RR	RR ≥ 1	MSCQ	<Daily Consumption
*Mylossoma* spp. (Pacú)	50	0.28 ± 0.41 (0.07–3.05)	64	2.3 ± 3.3 (0.6–24.4)	84	175 ± 93 (11–464)	92
*Pseudoplatystoma puntictifer* (Pintado)	4	1.54 ± 0.41 (1.05–2.01)	100	12.4 ± 3.3 (8.4–16.1)	100	22 ± 6 (16–30)	100
*Triportheus* spp. (Sardinha)	70	1.18 ± 0.71 (0.32–3.89)	100	9.4 ± 5.7 (2.6–31.1)	100	36 ± 19 (8–100)	100
*Psectrogaster rutiloides* (Branquinha)	135	0.39 ± 0.27 (0.11–2.07)	15	3.1 ± 2.1 (0.9–16.5)	99	105 ± 47 15–283)	100
*Prochilodus nigricans* (Curimatá)	44	0.61 ± 0.33 (0.06–1.20)	23	4.5 ± 6.1 (0.3–33.5)	93	101 ± 121 (27–542)	93
*Schizodon fasciatus* (Piau)	51	0.56 ± 0.77 (0.03–4.18)	43	4.9 ± 2.6 (0.5–9.6)	80	174 ± 186 (8–929)	84
*Cichla spp*. (Tucunaré)	23	4.46 ± 1.96 (1.97–9.45)	100	35.7 ± 15.7 (15.8–75.6)	100	9 ± 4 (3–16)	100
*Potamorhina spp.* (Chora)	123	0.52 ± 0.32 (0.09–1.51)	17	4.2 ± 2.5 (0.7–12.1)	97	91 ± 64 (21–342)	98
*Hypophthalmus marginatus* (Mapará)	13	1.44 ± 1.04 (0.29–3.75)	100	11.5 ± 8.4 (2.3–30.0)	100	41 ± 35 (9–112)	100

**Table 5:** Descriptive statistics for THg and MeHg concentrations in water, daily estimated ingestion, and risk assessment (mean ± standard deviation, minimum–maximum, and *n* = 53). The ADD and HQ are expressed in scientific notation due to their low values.

	Mean	SD	Min	Max
[THg] (ng L^−1^)	7.04	6.42	0.87	25.10
[MeHg] (ng L^−1^)	0.15	0.11	0.04	0.66
MeHg:THg (%)	4	5	0.5	24.5
Ingestion				
ADD–THg (µg kg^−1^)	1.66 × 10^−2^	1.52 × 10^−2^	2.06 × 10^−3^	5.93 × 10^−2^
ADD–MeHg (µg kg^−1^)	3.65 × 10^−4^	2.52 × 10^−4^	9.68 × 10^−5^	1.55 × 10^−3^
HQ–THg	4.16 × 10^−2^	3.79 × 10^−2^	5.16 × 10^−3^	1.48 × 10^−1^
HQ–MeHg	3.65 × 10^−3^	2.52 × 10^−3^	9.68 × 10^−4^	1.55 × 10^−2^

ADD: average daily dose. HQ: hazard quotient. The HQ was calculated in mg kg^−1^, but the ADD results are reported in μg kg^−1^ for a direct comparison with fish intake.

## Data Availability

The data referred to in this study are available upon request from the corresponding author.
